# Stress and Coping Strategies among Women in Late Motherhood

**DOI:** 10.3390/jcm13071995

**Published:** 2024-03-29

**Authors:** Mariola Mróz, Dominika Stobnicka, Agnieszka Marcewicz, Beata Szlendak, Grażyna Iwanowicz-Palus

**Affiliations:** 1 Chair of Obstetrics Development, Faculty of Health Sciences, Medical University of Lublin, 4-6 Staszica St., 20-081 Lublin, Poland; dominika.stobnicka@umlub.pl (D.S.); agnieszka.marcewicz@umlub.pl (A.M.); grazyna.iwanowicz-palus@umlub.pl (G.I.-P.); 2 Department of Midwifery, Centre of Postgraduate Medical Education, 01-004 Warsaw, Poland; beata.szlendak@cmkp.edu.pl

**Keywords:** late motherhood, stress, coping strategies

## Abstract

**Background:** The shifting reproductive age of women is reflected in European populations. Pregnancy in women older than 35 years is considered high-risk and can be an additional source of stress. The aim of this study was to assess the perceived stress of women experiencing late motherhood and the coping strategies used. **Methods:** The study was conducted in Poland by means of a diagnostic survey, using the COPE (Coping Orientation to Problems Experienced) Inventory, the Perceived Stress Scale (PSS), the Berlin Social Support Scales (BSSS), and a self-administered questionnaire. The study included 310 women who gave birth to their first child after the age of 35 and 313 respondents in a control group who gave birth before this age. **Results:** Based on the results, there were no statistically significant differences in feelings of stress among women who gave birth to their first child after the age of 35 (M = 18.33) compared to the control group (M = 18.14). However, statistically significant differences were observed regarding stress coping strategies. **Conclusions:** Women giving birth after the age of 35 were more likely to use strategies including active coping, planning, positive reformulation, acceptance, turning to religion, and seeking instrumental support.

## 1. Introduction

Stress is an inherent part of human life, being a reaction to the challenges with which humans are confronted [1,2,3]. The period of pregnancy, childbirth, and postpartum is a time for women to experience a variety of emotions. For the vast majority of women, these emotions are positive, but for some, pregnancy can be a source of stress as well as anxiety, worry, depression, and even irritability [4,5,6]. On the Social Readjustment Rating Scale (SRRS)—a life event scale that measures the amount of stress experienced—pregnancy and the arrival of a new family member received 40 and 39 LCU (*Life Change Unit Scores*) points, respectively, on the 100-point scale [7,8].

Recent studies in the literature use the term “*pregnancy-specific stress*”, which is differentiated from stress in general. The stress that a pregnant woman feels is mainly associated with adaptation difficulties and situations of everyday life. It very often hinders the process of adaptation to a new situation, as it alters the cognitive state in which the pregnant woman finds herself [9,10].

Perinatal anxiety and stress have a significant influence on the health situation of the mother and the development of the child. Due to their situation, pregnant women constitute a special group with regard to anxiety over their own health and that of their child. In this group of children, disorders of sleep, interaction with the mother, emotional development, and social relationships are more often observed. The consequences for the mother may be difficulties in breastfeeding and preterm labour [11].

Currently, there has been a big change in the perception of motherhood, as well as social and economic life, among women. Young women are increasingly interested in getting an education and developing a career, as well as achieving an adequate material status, which leads them to postpone the decision to have a child [12]. The analysis of socio-demographic data indicates that the number of births and fertility rates in Poland are steadily declining. The data gathered by *Statistics Poland* (formerly known as the *Central Statistical Office*) show a significant increase in births among women over the age of 35 over the past 20 years. In the year 2000, only 25% of all parturients in Poland were women over 30, while this number increased to 45% in 2015 [13,14]. The medical literature, in the case of women giving birth after the age of 35, uses the terms “advanced gestational age”, “mature older parturient”, and “late motherhood” [15].

Pregnancy is a physiological state leading to many changes in the functioning of a woman’s body, enabling the normal development of a foetus. Women’s bodies adapt more slowly to the changes posed by pregnancy and childbirth after the age of 35, with possible stresses from all systems and disorders in the course of labour. Therefore, pregnancy in women over the age of 35 is considered high-risk, which is an additional source of stress for these women [16,17,18].

What follows from the concept of stress is the idea of coping with stress. A difficult situation stimulates an individual to take specific action to achieve a balance between the demands of the environment and his or her abilities. An individual takes action to minimise the unpleasant sensations associated with stress and uses various coping methods [19,20]. Based on the traditional concept, coping with stress is a behaviour specific to a difficult situation. This behaviour should restore the balance between a person’s needs and predispositions [21]. The process of coping with stress involves some of the strategies available to the individual. The choice depends on the current situation which the person faces and his or her personality characteristics [22,23].

Late motherhood should be analysed with respect to a multitude of facets, since motherhood in mature women can lead to medical, psychological, and sociological problems [24]. Studies on the level of postpartum stress experienced by women make it possible to identify women who are particularly vulnerable to high levels of stress. They also make it possible to implement preventive measures, facilitating reductions in perceived anxiety and stress in mothers, which in turn can contribute to improving their mental states. Therefore, this provides a rationale for undertaking research on perceived stress and coping strategies of women in late motherhood.

## 2. Materials and Methods

This cross-sectional study was conducted in accordance with the guidelines for Strengthening the Reporting of Observational Studies in Epidemiology (STROBE). It was conducted by medical institutions in Poland. The criterion for selecting a hospital was the third and highest level of reference (the ability to provide top-quality specialist care for mothers and newborns). The study included 623 women staying in maternity wards of Lublin hospitals on the 2nd day after delivery, including 310 women who gave birth after the age of 35 and 313 subjects who gave birth before the age of 35.

The main objective of this study was to assess the level of perceived stress and coping strategies of women experiencing late motherhood. The specific objectives were to examine the relationship between the levels of perceived stress, coping strategies, and social support of women who gave birth after the age of 35 and to assess whether there was a relationship between perceived stress and the coping strategies and psychophysical condition of the respondents.

### 2.1. Study Design and Participants

The sample selection was purposive and non-probabilistic. The criteria for the inclusion of women in the experimental group included an age of 35 years or older, staying in the maternity ward on the second postpartum day, and being willing to participate in the study. The control group, on the other hand, consisted of women between the ages of 18 and 34 staying in the maternity ward on the second postpartum day who agreed to participate in the study.

#### 2.1.1. Data Collection

The study was approved by the Bioethics Committee of Lublin Medical University (KE-KE-0254/213/2016), as well as the managers and department heads in each hospital where the study was performed.

The respondents were informed of the anonymity of the study and that the results obtained would be used for scientific purposes. In total, 650 forms of prepared survey questionnaires were distributed (325 forms each for the experimental group and the control group). The consent forms and survey questionnaires were left in special boxes, which were opened after the survey was completed. Incorrectly and incompletely filled in questionnaires were not used in the analysis of the study. We received 623 correctly completed questionnaires (310 questionnaires were completed by the experimental group; the other 313 were completed by the control group). The return rate of all questionnaires that qualified for the study was 95.85% (95.38% for the experimental group and 96.31% for the control group).

#### 2.1.2. Assessments

The study used a diagnostic survey with questionnaires. The following research instruments were used: the Coping Orientation to Problems Experienced Inventory (Mini-COPE), the Berlin Social Support Scales (BSSS), the Perceived Stress Scale (PSS-10), and a standardised interview questionnaire.

#### 2.1.3. Instruments

**The Perceived Stress Scale (PSS-10)**—This questionnaire contains 10 questions to assess the intensity of stress created by life events over the past month. The respondent marks one answer on a scale (0—never, 1—almost never, 2—sometimes, 3—quite often, 4—very often). A higher number of points indicates a higher level of severity of perceived stress (0–10—low severity of stress, 10–20—average, 20–30—high, 30–40 very high). The Cronbach’s alpha coefficient is 0.86 [25].

**The Coping Orientation to Problems Experienced Inventory (Mini-COPE)**—This is a tool consisting of 28 statements comprising 14 strategies (there are 2 statements in each strategy). The frequency of use of certain strategies is expressed on a scale by marking one of the given answers to each statement (0—*I almost never do this*, 1—*I rarely do this*, 2—*I often do this*, 3—*I almost always do this*). Each scale is scored separately. The points from each scale are added up, and then the total is divided by 2. The scores are placed in a range from 1 to 3. The higher the score, the more often the respondents used a particular strategy. Strategies such as planning, seeking instrumental support, and active coping are categorised as problem-focused strategies. Adaptive strategies include acceptance and positive reframing, which reduce tension and negative emotions. Emotion-focused strategies include seeking emotional support, denial, and turning to religion. In contrast, self-distraction, venting, blaming oneself, behavioural disengagement, use of psychoactive substances, and humour are avoidance strategies. The Cronbach’s alpha coefficients for each scale range from 0.48 to 0.94 [26].

**Berlin Social Support Scales (BSSS)**—These are questionnaires used to measure cognitive and behavioural dimensions of social support. The subscales used for the study were: perceived available emotional support, seeking support, and currently receiving support. The women surveyed marked answers from 1 to 4 (where 1, in the opinion of the respondents, is a completely false statement, while 4 is a completely true one). The sum of the mean values for each scale was used in the study. The degree of intensity of social support depends on the number of points obtained. The value of the Cronbach’s alpha coefficient is 0.80 [26,27].

**Authors’ own survey questionnaire**—This questionnaire takes into account the characteristics of the women surveyed (age, education, residence, relationship status, and self-reported financial status) and questions concerning the subject (physical condition and mental condition).

### 2.2. Statistical Analysis

The collected study material was analysed using the IBM SPSS Statistics package (version 21). Quantitative variables were described using means (Ms), medians (Mes), and standard deviations (SDs). Qualitative variables were presented using numbers (n) and percentages (%) for each category. In a situation in which the assumptions for parametric tests (variables measured at the quantitative level of measurement) were met, the Student’s *t*-test for independent groups was used to verify the hypothesis of the equality of the means of the variable studied in the two populations. For comparisons between more than two independent groups, the Kruskal–Wallis ANOVA on ranks (H) was used. In order to determine the influence of several variables (predictors) on the dependent variable, a multivariate regression analysis was conducted using the stepwise method. The dependent variable was the level of perceived stress (PSS-10). In turn, the independent variables were stress coping strategies, the generalised self-efficacy scale, and social support received. The results were considered statistically significant at *p* < 0.05.

## 3. Results

Table 1 shows the respondents’ characteristics. The experimental group consisted of women over the age of 35 (49.75%), while the control group consisted of women under the age of 35 (50.25%) (Table 1).

The majority of respondents in both groups had received tertiary education, were urban residents, were in a relationship, and rated their socioeconomic status as good (Table 1).

In both groups of respondents, there was a predominance of those who evaluated their mental and physical condition and quality of life as good. In the control group, the evaluation of the psycho-physical condition and quality of life was higher compared to women experiencing late motherhood (*p* < 0.05) (Table 1).

Among women who gave birth after the age of 35 (M = 18.33) and postpartum women in the control group (M = 18.14), there were no statistically significant differences in perceived stress (*p* < 0.633) (Table 2).

However, statistically significant differences were found between the experimental group and the control group in stress coping strategies (*p* < 0.05). Strategies such as acceptance (M = 1.82) and denial (M = 0.83) were used more frequently among women who gave birth after the age of 35 compared to respondents in the control group (acceptance: M = 1.69, denial: M = 0.70) (Table 2).

There were no differences between the experimental groups in terms of social support (*p* > 0.05) (Table 2).

The results showed that the higher the values of perceived stress in women in the experimental group (M = 20.83) and in the control group (M = 19.78), the lower their physical condition score (*p* < 0.001) (Table 3).

Respondents who gave birth after the age of 35 declaring very good physical condition were statistically significantly more likely (*p* < 0.05) to choose the following strategies: active coping (M = 2.46), positive reframing (M = 1.77), and acceptance (M = 1.99). The analysis of the survey results also shows that strategies such as denial (M = 0.98), behavioural detachment (M = 0.97), and blaming oneself (M = 1.53) are more often preferred by respondents with an average physical condition (*p* < 0.05) (Table 3).

The analysis of the results also showed significant statistical differences (*p* < 0.05) between the stress coping styles and physical condition of the respondents who were in the control group. The higher the physical condition score of the respondents, the more often the women chose the strategy of active coping (M = 2.32) and seeking emotional support (M = 2.20), while at the same time they were least likely to use self-distraction (M = 1.38) (Table 3).

In terms of the relationship between the level of perceived stress and the assessment of mental condition, statistically significant correlations were shown in the group of women who gave birth at over 35 years of age (*p* < 0.001), as well as in the group of younger women (*p* < 0.001). Respondents with the highest values of perceived stress rated their mental condition as average (experimental group M = 22.44, control group M = 22.97) (Table 4).

The results indicate that the higher the mental condition score of the respondents in the experimental group, the more frequently the following strategies were used: active coping (M = 2.28), planning (M = 2.13), positive reframing (M = 1.81), acceptance (M = 1.96), turning to religion (M = 1.68), and seeking instrumental support (M = 1.94), (*p* < 0.05). In contrast, strategies such as denial (M = 1.18), substance abuse (M = 0.44), behavioural disengagement (M = 1.20), and blaming oneself (M = 1.72) were used most frequently among respondents rating their mental condition as average (Table 4).

Among women representing the control group, the results show that respondents who rated their mental condition as very good were significantly more likely to use strategies such as active coping (M = 2.24), planning (M = 2.06), positive reframing (M = 1.75), and seeking emotional support (M = 2.13), compared to women who rated their mental condition as good or average (*p* < 0.05). In contrast, strategies such as denial (M = 0.88), psychoactive substance use (M = 0.51), behavioural disengagement (M = 0.95), and blaming oneself (M = 1.76) were used most often in the group of women surveyed who rated their mental condition as average (Table 4).

In order to determine the influence of several variables (predictors) on the dependent variable, a multivariate regression analysis was conducted using the stepwise method. The dependent variable was the level of perceived stress as measured by the PSS-10. In turn, the independent variables were stress coping strategies, the generalised self-efficacy scale, and social support received (Table 5).

The proposed regression model was found to fit the data well (F = 39.729, df = 9.613, *p* < 0.001). The results obtained from the model show that perceived stress positively correlates with the following coping strategies: venting (ß = 0.156, *p* < 0.001), denial (ß = 0.086, *p* = 0.018), and blaming oneself (ß = 0.088, *p* = 0.021). In contrast, a negative correlation was shown between perceived stress and the strategies of seeking emotional support (ß = −0.122, *p* = 0.001) and positive reframing (ß = −0.090, *p* = 0.009) (Table 5).

In addition, perceived stress is negatively correlated with perceived emotional support (ß = −0.104, *p* = 0.018) and with currently received support (ß = −0.081, *p* = 0.045). In contrast, a higher level of support seeking correlates positively with perceived stress (ß = 0.082, *p* = 0.027). The constant value was statistically significant (*p* < 0.001). The model tested explains 37% of the variation in the dependent variable (PSS-10) (Table 5).

## 4. Discussion

Motherhood is one of the most important and unique experiences in a woman’s life. However, more and more women choose to delay having children. The phenomenon known as late motherhood is associated with certain challenges, both biological and psychosocial in their nature. Understanding the stress levels and coping strategies of women in late motherhood is not only important from the perspective of the mental and physical health of these women, but it also holds social and scientific significance [28,29,30].

Analysing these aspects can help identify specific challenges related to late motherhood. Studying the levels of perceived stress and coping strategies can contribute to improving healthcare directed towards these women, addressing both mental and physical health. Delving into these issues can lead to the development of knowledge regarding the impact of delayed motherhood on various aspects of women’s lives. Understanding the stress and coping strategies of women in late motherhood is essential for developing appropriate supportive measures for them.

The regression analysis conducted for this study showed that perceived stress was positively correlated with the use of strategies such as venting, denial, and self-blame and negatively correlated with strategies like acceptance and positive reframing. Based on the results, it was also found that women experiencing late motherhood were more likely to use strategies to reduce tension and negative emotions compared to younger women. The analysis of the study by Zuralska et al. showed that pregnant women often choose active coping and planning strategies in stressful situations. These attitudes allow them to better cope with current stress and prove to be the most effective ways of coping with difficult situations [31].

Zanardo et al. showed that respondents in a group of pregnant women under the age of 35 with normal gestation were most likely to use a task-oriented style, followed by an emotion-oriented style in stressful situations. Avoidance-oriented, attention-diverting, and distraction-oriented coping strategies were the least frequently used [32]. Among women with complicated pregnancies, Murlikiewicz and Sieroszewski noted that pregnant women most often chose emotion-focused strategies. The authors also observed that emotion-focused pregnant women tended to focus on subjective emotional experiences and engaged in substitution-based activities [33]. Borcherding and Rutkowska et al., studying pregnant women in the third trimester of pregnancy, found similar results. They showed that the most frequently used coping strategy in stressful situations was problem-focused, while avoidance methods were used least frequently [34,35]. In a stressful situation, the women took action to solve the problem at hand. In addition, Rutkowska et al. noted that the emergence of negative emotions in an at-risk pregnancy is inevitable. However, using proactive strategies, pregnant women were able to adapt to the new situation and take constructive action that led to the solution of the problem [35]. Zuralska et al., studying postpartum women, also confirmed that the “active coping” strategy was the method preferred by postpartum women. In contrast to those who choose avoidance strategies, women using active coping reported feeling less tired and burdened by new responsibilities [31]. Thus, it is worth emphasising the importance of these strategies in the context of coping with difficulties and stress during pregnancy and after childbirth.

The issue of stress is linked to religiosity and social support, which can influence the way individuals cope with stress and experience quality of life.

Social support is understood as a type of social interaction undertaken in problematic or difficult situations. The scientific literature indicates that the social support that a woman receives during pregnancy as well as after childbirth has a significant impact on reducing levels of perceived stress, negative emotions, and anxiety and positively influences the course of pregnancy [27,36,37,38,39,40].

In terms of social support, no significant differences were observed between women experiencing late motherhood and women who gave birth to their first child at a younger age. There was also a negative correlation between perceived stress and perception of emotional support and currently received support and a positive correlation between the level of perceived stress and social support seeking behaviour.

In his research, Riberio proved that positive social support can significantly affect the way pregnant women cope with stressful situations, enabling them to adapt more quickly to their roles as mothers. One of the key sources of this support is family [41]. MaDogmei’s findings also suggest that emotional support from family members, especially the spouse, can mitigate the adverse effects of perinatal stressors, increasing the new mother’s confidence and thereby reducing her stress levels [42].

Kossakowska showed that among women in very good mental condition, no statistically significant correlations were found between the choice of stress coping strategy and the level of social support. However, among women with average mental condition, strong correlations were found between social support received and avoidance strategies. A higher rating of support received was associated with a higher frequency of taking active measures in stress coping situations [21].

In contrast, Giurgescu et al. observed that prayer was the most frequently used prenatal coping strategy among a group of high-risk pregnant women who reported low levels of uncertainty, moderate levels of distress, and high levels of social support, while avoidance was the least frequently used strategy. Women who reported higher levels of uncertainty reported less social support, poorer psychological well-being, less positive reframing, and more frequent use of avoidance. Avoidance significantly mediated the effect of uncertainty on psychological well-being. Social support had a significant direct effect on preparation for motherhood [43].

Both the impact of stress and coping strategies are important for women’s mental and physical health. According to an analysis by Stadnicka et al., pregnant women under the age of 25 rated their physical condition as very good. However, those surveyed over the age of 35 indicated average physical form [44]. During the analysis of our study, it was noted that one in three women over the age of 35 rated her physical condition as average. This fact was observed statistically significantly more frequently than in younger women. The analysis of the survey results also indicates that strategies such as denial, behavioural disengagement, and blaming oneself are more often preferred by respondents with average physical condition. Women who gave birth after the age of 35 and who also reported very good physical condition were more likely to use problem-solving-focused adaptive strategies and techniques to reduce stress and negative emotions, such as active coping, positive reframing, and acceptance, than those with lower stress levels.

Statistical analyses of the relationship between the level of perceived stress and ratings of psychological conditions showed significant correlations both among women who gave birth after the age of 35 and among younger mothers. Respondents with the highest values of perceived stress rated their mental condition as average. As the rating of women’s mental condition increased in the experimental group, a more frequent use of adaptive, emotion-focused, and problem-focused strategies was observed. In contrast, those describing their mental condition as average were most likely to choose avoidance and emotion-focused strategies.

The literature review indicates that adequate social support plays a protective role against the effects of stress [45,46,47,48].

Social support, an important determinant of health and well-being, is the most effective way to cope with severely stressful situations. Receiving social support positively influences positive experiences and life satisfaction, so the need to care for women in the puerperium, especially those experiencing additional stresses, such as late motherhood and related difficulties, should be given special consideration. Plotting the types of behaviour in the face of stress, women experiencing late motherhood can form the basis for interventions aimed at improving stress management, undertaken both during follow-up visits by the doctor and midwife, as well as in antenatal classes, thereby minimising the negative consequences of stress.

The literature points to the significant impact of stress and anxiety on human existence. These factors can also notably influence a mother’s life, who is burdened with anxiety, faces difficulties in coping with the challenges of motherhood, is excessively concerned for the child, and struggles in making decisions regarding their upbringing and care [45,49].

The educational and informational aspect of the psycho-physical condition and the social consequences of choosing motherhood at an advanced age is extremely important. It is very important to take measures to reduce the level of anxiety and stress experienced by women in order to increase their psychological well-being and quality of life. These measures will further contribute to reducing the risk of perinatal complications and psychological disorders.

## 5. Conclusions

Women giving birth after the age of 35 tended to use strategies including active coping, planning, positive reframing, acceptance, turning to religion, and seeking instrumental support. There was a significant correlation between the level of stress, coping strategies, and social support of the women studied. 

It is essential to implement preventive measures that contribute to reducing feelings of anxiety and stress in mothers and improving their skills in coping with stress.

### Strengths and Limitations of This Study

Our study used a standardised instrument, allowing other researchers to compare results and monitor changes. The strengths of this study also include its large sample size and personal communication with each respondent.

As for the limitations, it should be pointed out that this was a cross-sectional study; therefore, causal relationships could not be established.

Both late motherhood and stress during pregnancy are extensive topics. Various factors, such as obstetric history, can influence the stress levels in women experiencing late motherhood. Therefore, further research exploration of this topic is needed.

## Figures and Tables

**Table 1:** Participants’ characteristics.

Participants’ Characteristics		Experimental Group		Control Group	
		*n*	%	*n*	%
Age	≤34 y/o	-	-	313	50.25
	≥35 y/o	310	49.75	-	-
Education	High school education	72	23.2	84	26.8
	College/university	238	76.8	229	73.2
Residence	Urban	244	78.7	171	54.7
	Rural	66	21.3	142	45.4
Relationship status	Married	288	92.9	301	96.2
	Single	22	7.1	12	3.8
Self-reported financial standing	Very good	84	27.1	73	23.3
	Good	180	58.1	197	62.9
	Bad	46	14.8	43	13.7
Physical condition	Very good	37	11.9	47	15.0
	Good	178	57.4	201	64.2
	Average	95	30.6	65	20.8
Mental condition	Very good	67	21.6	97	31.0
	Good	181	58.4	177	56.5
	Average	62	20.0	39	12.5
Quality of life	Very good	44	14.2	72	23.0
	Good	219	70.6	219	70.0
	Average	47	15.2	22	7.0

**Table 2:** Relationship analysis of perceived stress levels, coping strategies, and social support in the study and control groups.

	Factors		M	Me	SD	*p*
PSS–10	Perceived Stress Scale	*Study group*	18.33	19.00	6.30	0.633
		*Control group*	18.14	18.00	6.02	
Mini-COPE	Active coping	*Study group*	2.12	2.00	0.65	0.861
		*Control group*	2.11	2.00	0.64	
	Planning	*Study group*	2.06	2.00	0.62	0.067
		*Control group*	1.96	2.00	0.63	
	Positive reframing	*Study group*	1.66	1.50	0.67	0.833
		*Control group*	1.65	1.50	0.65	
	Acceptance	*Study group*	1.82	2.00	0.59	0.009
		*Control group*	1.69	2.00	0.66	
	Humour	*Study group*	0.76	0.50	0.65	0.610
		*Control group*	0.78	1.00	0.56	
	Religion	*Study group*	1.50	1.50	0.90	0.273
		*Control group*	1.40	1.00	1.31	
	Emotional support	*Study group*	1.93	2.00	0.64	0.256
		*Control group*	1.99	2.00	0.61	
	Instrumental support	*Study group*	1.85	2.00	0.63	0.854
		*Control group*	1.86	2.00	0.64	
	Self-distraction	*Study group*	1.55	1.50	0.67	0.958
		*Control group*	1.55	1.50	0.64	
	Denial	*Study group*	0.83	1.00	0.69	0.012
		*Control group*	0.70	0.50	0.58	
	Venting	*Study group*	1.30	1.50	0.64	0.709
		*Control group*	1.29	1.50	0.62	
	Substance use	*Study group*	0.29	0.00	0.54	0.102
		*Control group*	0.22	0.00	0.48	
	Behavioural disengagement	*Study group*	0.80	1.00	0.63	0.053
		*Control group*	0.71	0.50	0.62	
	Self-blame	*Study group*	1.27	1.00	0.70	0.151
		*Control group*	1.19	1.00	0.73	
BSSS	Perceived available emotional support	*Study group*	3.58	3.75	0.50	0.377
		*Control group*	3.54	3.75	0.51	
	Support seeking	*Study group*	3.10	3.00	0.64	0.181
		*Control group*	3.03	3.00	0.65	
	Actually received support	*Study group*	3.49	3.67	0.42	0.243
		*Control group*	3.53	3.73	0.42	

M—mean, Me—median, SD—standard deviation.

**Table 3:** Analysis of the relationship between the impact of perceived stress and coping strategies and physical condition in the study and control groups.

Factors			Physical Condition						*p*
			Very Good		Good		Average		
			M	SD	M	SD	M	SD	
PSS-10	Perceived Stress Scale	*Study group*	14.30	6.12	17.83	5.84	20.83	6.22	<0.001
		*Control group*	15.04	6.04	18.33	5.79	19.78	5.98	<0.001
Mini-COPE	Active coping	*Study group*	2.46	0.64	2.12	0.67	1.98	0.59	<0.001
		*Control group*	2.32	0.58	2.06	0.66	2.10	0.60	0.039
	Planning	*Study group*	2.23	0.52	2.07	0.61	1.96	0.65	0.238
		*Control group*	2.02	0.68	1.95	0.60	1.96	0.69	0.672
	Positive reframing	*Study group*	1.77	0.64	1.72	0.67	1.52	0.65	0.027
		*Control group*	1.81	0.63	1.65	0.65	1.54	0.67	0.155
	Acceptance	*Study group*	1.99	0.57	1.89	0.58	1.61	0.56	<0.001
		*Control group*	1.72	0.67	1.69	0.65	1.66	0.72	0.803
	Humour	*Study group*	0.81	0.68	0.81	0.65	0.65	0.62	0.112
		*Control group*	0.80	0.73	0.80	0.53	0.74	0.52	0.790
	Religion	*Study group*	1.49	0.81	1.51	0.91	1.47	0.92	0.925
		*Control group*	1.50	1.06	1.35	0.96	1.49	2.16	0.561
	Emotional support	*Study group*	2.04	0.57	1.91	0.67	1.92	0.58	0.620
		*Control group*	2.20	0.55	1.94	0.62	1.96	0.57	0.017
	Instrumental support	*Study group*	1.82	0.64	1.85	0.67	1.88	0.55	0.965
		*Control group*	1.96	0.60	1.83	0.66	1.91	0.60	0.571
	Self-distraction	*Study group*	1.43	0.74	1.59	0.70	1.51	0.60	0.112
		*Control group*	1.38	0.69	1.62	0.61	1.43	0.68	0.006
	Denial	*Study group*	0.64	0.61	0.80	0.67	0.98	0.75	0.036
		*Control group*	0.61	0.64	0.70	0.54	0.78	0.65	0.289
	Venting	*Study group*	1.28	0.79	1.24	0.60	1.44	0.64	0.107
		*Control group*	1.22	0.62	1.26	0.62	1.40	0.62	0.270
	Substance use	*Study group*	0.14	0.33	0.31	0.56	0.31	0.55	0.220
		*Control group*	1.22	0.62	1.26	0.62	1.40	0.62	0.270
	Behavioural disengagement	*Study group*	0.53	0.56	0.77	0.63	0.97	0.62	0.001
		*Control group*	0.78	0.89	0.67	0.54	0.75	0.59	0.652
	Self-blame	*Study group*	0.97	0.51	1.20	0.67	1.53	0.74	<0.001
		*Control group*	0.97	0.64	1.20	0.74	1.32	0.75	0.059

M—mean, SD—standard deviation.

**Table 4:** Analysis of the relationship between the impact of perceived stress and coping strategies and mental condition in the study and control groups.

Factors			Mental Condition						
			Very Good		Good		Average		*p*
			M	SD	M	SD	M	SD	
PSS-10	Perceived Stress Scale	*Study group*	14.91	5.55	18.18	5.70	22.44	6.47	<0.001
		*Control group*	15.42	5.72	18.56	5.60	22.97	5.10	<0.001
Mini-COPE	Active coping	*Study group*	2.28	0.63	2.15	0.66	1.83	0.59	<0.001
		*Control group*	2.24	0.67	2.06	0.63	1.96	0.57	0.009
	Planning	*Study group*	2.13	0.56	2.09	0.64	1.87	0.58	0.032
		*Control group*	2.06	0.71	1.96	0.56	1.74	0.70	0.044
	Positive reframing	*Study group*	1.81	0.67	1.71	0.65	1.36	0.63	<0.001
		*Control group*	1.75	0.75	1.69	0.57	1.26	0.62	<0.001
	Acceptance	*Study group*	1.96	0.52	1.83	0.60	1.63	0.59	0.004
		*Control group*	1.71	0.72	1.71	0.63	1.51	0.64	0.216
	Humour	*Study group*	0.85	0.66	0.73	0.62	0.74	0.71	0.420
		*Control group*	0.78	0.60	0.81	0.52	0.65	0.63	0.149
	Religion	*Study group*	1.68	0.92	1.39	0.88	1.62	0.90	0.028
		*Control group*	1.39	1.02	1.44	1.51	1.24	0.97	0.759
	Emotional support	*Study group*	1.99	0.56	1.94	0.67	1.85	0.62	0.281
		*Control group*	2.13	0.65	1.96	0.56	1.73	0.63	0.003
	Instrumental support	*Study group*	1.94	0.58	1.86	0.65	1.73	0.63	0.040
		*Control group*	1.88	0.73	1.88	0.57	1.73	0.71	0.288
	Self-distraction	*Study group*	1.52	0.71	1.52	0.69	1.65	0.59	0.554
		*Control group*	1.51	0.69	1.58	0.61	1.49	0.65	0.367
	Denial	*Study group*	0.63	0.50	0.79	0.70	1.18	0.74	<0.001
		*Control group*	0.51	0.55	0.77	0.54	0.88	0.71	<0.001
	Venting	*Study group*	1.25	0.67	1.27	0.63	1.48	0.64	0.091
		*Control group*	1.20	0.68	1.29	0.58	1.49	0.61	0.063
	Substance use	*Study group*	0.14	0.33	0.29	0.55	0.44	0.63	0.009
		*Control group*	0.17	0.39	0.19	0.44	0.51	0.70	0.001
	Behavioural disengagement	*Study group*	0.65	0.55	0.72	0.62	1.20	0.58	<0.001
		*Control group*	0.65	0.75	0.68	0.52	0.95	0.60	0.007
	Self-blame	*Study group*	1.03	0.58	1.21	0.66	1.72	0.73	<0.001
		*Control group*	1.03	0.71	1.15	0.69	1.76	0.77	<0.001

M—mean, SD—standard deviation.

**Table 5:** Regression analysis of PSS-10 and stress coping strategies and social support received.

Predictor	B	*ß*	t	*p*
Constant	38.795		17.037	<0.001
Venting (Mini-COPE)	1.522	0.156	4.408	<0.001
Emotional support (Mini-COPE)	−1.208	−0.122	−3.295	0.001
Denial (Mini-COPE)	0.823	0.086	2.370	0.018
Perceived available emotional support (BSSS I EMO)	−1.155	−0.104	−2.378	0.018
Positive reframing (Mini-COPE)	−0.836	−0.090	−2.638	0.009
Self-blame (Mini-COPE)	0.753	0.088	2.321	0.021
Support seeking (BSSS III)	0.786	0.082	2.213	0.027
Actually received support (BSSS IV)	−1.184	−0.081	−2.012	0.045

## Data Availability

Data are available upon reasonable request.
